# Our Future

**Published:** 1886-01

**Authors:** 


### HALL'S JOURNAL OF HEALTH

Vol. 33. JANUARY, 1886. No. 1.

572

### ANNOUNCEMENT.

### OUR FUTURE.

The last number of the Journal contained an announcement of the new arrangement under which its publication will henceforth be conducted. It is the design of the publisher to give its patrons the benefit of well attested cures by whatever means or system of treatment; hence, the Journal will not be permitted to become the especial organ of any one school of medicine to the exclusion of others. With the editorial department under control of a single mind, however liberally inclined in other ways, if trained in one or the other of these established schools, there will be almost invariably, a strong leaning towards the one which he has adopted, and with it a tendency to uphold it by depreciating others, however meritorious. To guard against this, it is deemed advisable to dispense, so far as practicable, with the presentation of *theories* and give our readers the benefit of facts which can be relied upon, having relation to the preservation of health and the cure of diseases.

It is our design to make the JOURNAL, in the truest sense, a *family* publication, giving sufficient valuable information in every number to more than compensate for the cost of a year's subscription. Nothing that we eat, drink, breathe and wear but has a direct bearing upon life and health, and life without health is little else than a burden. Not only this, but the method of preparing food for the table, times and manner of eating, sleeping and recuperating impared energy, have more to do with the preservation of health than the generality of people understand. But we have come to know that no general rule can be laid down equally adapted to all persons, even those of like temperament and corresponding physique, whose habits and mode of life essentially differ.

Our home department, under the immediate supervision of able and experienced writers, will deal with these questions in a manner which cannot fail to interest and oftentimes instruct the members of the family circle. It is with the design of giving prominence to this department and others of equal value, that the reading matter of the JOURNAL has been enlarged, a feature which we make no doubt our patrons, past and present, will not fail to appreciate. We wish them one and all a prosperous and happy new year.

